# Acupuncture for Crohn’s disease: protocol for a systematic review and meta-analysis of randomized clinical trials

**DOI:** 10.1097/MD.0000000000032163

**Published:** 2022-12-02

**Authors:** Jiazhen Cao, Qianhui Yu, Mengmeng Sun, Min He, Renming Liu, Wu Liu, Fuchun Wang, Tie Li

**Affiliations:** a Acupuncture and Moxibustion Academy, Changchun University of Chinese Medicine, Changchun, China; b Northeast Asian Institute of Traditional Chinese Medicine, Changchun University of Chinese Medicine, Changchun, China; c Department of Acupuncture, The Affiliated Hospital of Changchun University of Chinese Medicine, Changchun, China.

**Keywords:** acupuncture, Crohn’s disease, protocol, systematic evaluation

## Abstract

Crohn’s disease (CD) is a chronic recurrent gastrointestinal disorder with a high incidence of inflammation due to intestinal inflammation. Acupuncture is commonly used as an alternative therapy for patients with CD. The aim of this study was to design a systematic review and meta-analysis protocol, to provide guidance for the establishment of systematic evaluation and meta-analysis on the efficacy and safety of acupuncture on CD.

**Methods:**

We will search PubMed, the Cochrane Library, Embase, Web of Science, and 4 Chinese databases: China National Knowledge Infrastructure (CNKI), Wanfang database, VIP database, and Chinese Biomedical Database to obtain randomized controlled trials of CD treated with acupuncture from inception to November 5, 2022. Primary outcome include CD symptoms severity and clinical efficacy, secondary outcome indicators include laboratory indicators or inflammatory markers, severity of endoscopic lesions, quality of life, and safety outcomes. We will analyze the data using RevMan V.5.4 software. Two reviewers will assess the risk of bias and study quality by the Cochrane Collaboration Risk of Bias Tool and GRADE methods, respectively.

**Results:**

This systematic review and meta-analysis protocol will analyze the efficacy, symptom improvement, quality of life, and safety of acupuncture therapy for CD.

**Conclusion:**

This protocol outlines the planned scope and methodology of a forthcoming systematic review and meta-analysis to provide guidelines for a rigorous assessment of the efficacy and safety of acupuncture for the treatment of CD.

## 1. Introduction

Crohn’s disease (CD) is a chronic, recurrent inflammatory disease caused by a combination of genetic, immune, microbial, and environmental factors that can occur anywhere in the gastrointestinal tract, with inflammation involving all layers of the gastrointestinal tract.^[1]^ The main symptoms individuals with CD is diarrhea, abdominal pain, bloody stools, weight loss, and malnutrition. It can also cause a variety of gastrointestinal tract, extra-gastrointestinal tract complications, and systemic manifestations.^[2]^ Over the past 3 decades, the incidence of CD has gradually increased at a rate of 4% to 15% per year globally.^[3]^ In recent years, the rapid development of newly industrialized countries such as China has led to increasingly younger age of onset of CD. According to a recent large multicenter clinical study conducted in China reported that average age of CD onset is 32.3 years old.^[4]^ The pathogenic mechanism of CD is directly related to specific factors such as genetics, abnormal intestinal immunity, and dysfunctional intestinal flora.^[1]^ The most widely used drugs in CD (including corticosteroids, immunosuppressants, and biologics) is rather unsatisfactory and some of them are costly and have multiple side effects.^[5]^ In approximately one-third to two-thirds of patients, the most effective drugs for CD, such as TNF-α antagonists, do not respond or become less effective over time.^[6]^ Therefore, it is imperative to find an effective, safe, and side-effect-free complementary alternative therapy for CD.

Acupuncture is a widely used complementary alternative therapy for inflammatory bowel disease worldwide.^[7]^ Acupuncture has been found to reduce CD activity index (CDAI), improve quality of life and general well-being in patients with mild and moderate CD, as well as improve general patient health and reduce concentrations of serum a1 acidic glycoprotein16.^[8]^ And acupuncture combined with moxibustion can also significantly reduces C-reactive protein levels and increases hemoglobin levels.^[9]^ The effect of acupuncture in the treatment of CD may be related to increased intestinal anti-inflammatory bacterial abundance, enhancement of the intestinal barrier, and regulation of Th1/Th17-related cytokine circulation.^[10]^ Although the above clinical and mechanistic studies suggest that acupuncture can alleviate CD symptoms, however, there is still a lack of relevant systematic reviews and meta-analysis synthesizing the clinical efficacy and safety of acupuncture for CD. Standard protocol is needed prior of the systematic reviews and meta-analysis. In summary, it is urgent to strictly synthesize the evidence regarding the efficacy and safety of acupuncture for CD. Therefore, this protocol outlines the planned scope and methodology of a forthcoming systematic review and meta-analysis to provide guidelines for a rigorous assessment of the efficacy and safety of acupuncture for the treatment of CD. It is also expected to provide new research treatment-guided decisions for the treatment of CD with acupuncture.

## 2. Methods

### 2.1. Protocol registration

This protocol was registered in PROSPERO (CRD42021266406). It will be followed the standard methods of systematic review and meta-analysis. It will adhere to the Preferred Reporting Items for systematic reviews and meta-analyses reporting guidelines (see online supplemental appendix 1).

### 2.2. Study type

In this protocol design, we will design a systematic review and meta-analysis, in which randomized controlled trials articles on CD disease treated by acupuncture will be collected to assess the clinical efficacy, quality of life, symptom improvement, and safety of acupuncture for CD. In addition, case series, observational studies and retrospective studies, qualitative studies, animal studies, and review articles will be excluded. Some quasi-randomized controlled trials (such as allocating by alphabetical order, alternate days of the week or date of birth) are also excluded. Crossover trials will be excluded because of the potential for legacy effects. All eligible trials will be included, with no restrictions on study area, race, patient age, or gender.

### 2.3. Participants type

This review will include patients with CD at the time of study entry, as defined by a recognized CDAI, endoscopy, histopathology, or radiology, without other limitations, such as age, gender, or race.^[11]^

### 2.4. Interventions type

In the intervention group, patients received acupuncture as the sole intervention or primary treatment (including acupoint-based traditional hand acupuncture, electroacupuncture, warm acupuncture, or laser acupuncture) in combination with other interventions (including traditional medicine or other available Chinese medical practices). In the control group, patients received placebo, or conventional treatment (such as aminosalicylic acid, glucocorticoids, immunomodulators, and biologics) or no treatment.. Other interventions should be the same in both groups.

### 2.5. Outcome measures

1) The primary outcomes include:
  a) The symptoms severity of CD disease, such as CDAI score and serum C-reactive protein, it is used to assess and can also be used to assess^[12]^;
  b) The efficacy of treatment, such as the proportion of patients with clinical remission at the completion of treatment, generally divided into 3 levels (clinical cure, effectiveness, and invalid) according to different syndrome improvement degrees.
2) The secondary outcomes include:
  1. Laboratory indicators or inflammatory markers related to CD: hemoglobin, such as diamine oxidase, lipopolysaccharides, d-lactic acid, IFN-γ, IL-17A, IL-23, TNF-α, and IL-1β;
  2. Severity of endoscopic lesions, such as CD endoscopic index of severity and simple endoscopic score for Crohn’s disease;
  3. Quality-of-life measured by standardized questionnaires with established validity, such as the 12-Item Short Form Health Survey, 36-Item Short Form Health Survey;
  4. Safety outcomes, incidence of adverse events such as skin breakdown at the acupuncture site, bleeding, hematoma, unbearable pain, infection, and shock.

### 2.6. Search strategy

We searched 4 international electronic databases: PubMed, the Cochrane Library, Embase, Web of Science, and 4 Chinese databases: China National Knowledge Infrastructure, Wanfang database, VIP database, and Chinese Biomedical Database. The search period is from the date of database creation to November 5, 2022. The language of the trial studies was limited to Chinese or English. The type of study was a clinical randomized controlled trial. When searching the English database, the 3 key components of intervention method, disease and study type were used: (“acupuncture” or “needling” or “acupressure” or “acupoint*” or “MA” or “electroacupuncture” or “electro-acupuncture” or “EA” or “meridian*”) AND (“Crohn Disease” or “Crohn’s Enteritis” or “Regional Enteritis” or “Crohn’s Disease” or “Crohns Disease” or “ Granulomatous Enteritis” or “ileocolitis” or “Granulomatous Colitis” or “Terminal ileitis” or “Regional lleitides” or “Regional ileitis” or “CD”) AND (“randomized controlled trials” or “case-control studies” or “observational studies” or “case series” or “trial”). The Chinese databases were searched using Chinese characters with the same meaning. A detailed search strategy for the PubMed database is provided as shown in Table 1. The same or adapted search strategies will then be used for other electronic databases. In addition, reference lists of relevant original studies will be screened to identify additional potential citations.

**Table 1:** The search strategy for PubMed database.

Number	Search terms
#1	Acupuncture OR Acupuncture Therapy OR Acupuncture Points [MeSH]
#2	acupuncture[Title/Abstract] OR needling [Title/Abstract] OR acupressure[Title/Abstract] OR acupoint*[Title/Abstract] OR MA[Title/Abstract] OR electroacupuncture[Title/Abstract] OR electro*-acupuncture [Title/Abstract] OR EA[Title/Abstract] OR meridian*[Title/Abstract]
#3	#1 OR #2
#4	Crohn’s disease [MeSH]
#5	Crohn’s Enteritis [Title/Abstract] OR Regional Enteritis [Title/Abstract] OR Crohn’s disease [Title/Abstract] OR Crohns Disease [Title/Abstract] OR Granulomatous Enteritis [Title/Abstract] OR ileocolitis [Title/Abstract] OR Granulomatous Colitis [Title/Abstract] OR Terminal ileitis [Title/Abstract] OR Regional lleitides [Title/Abstract] OR Regional ileitis [Title/Abstract] OR CD [Title/Abstract]
#6	#4 OR #5
#7	randomized controlled trial [MeSH]
#8	randomized controlled trial [Title/Abstract] OR a case-control studies [Title/Abstract] OR observational studies [Title/Abstract] OR case series [Title/Abstract] OR trial [Title/Abstract]
#9	#7 OR #8
#10	#3 AND #6 AND #9

### 2.7. Study selection and data extraction

**Figure 1.**
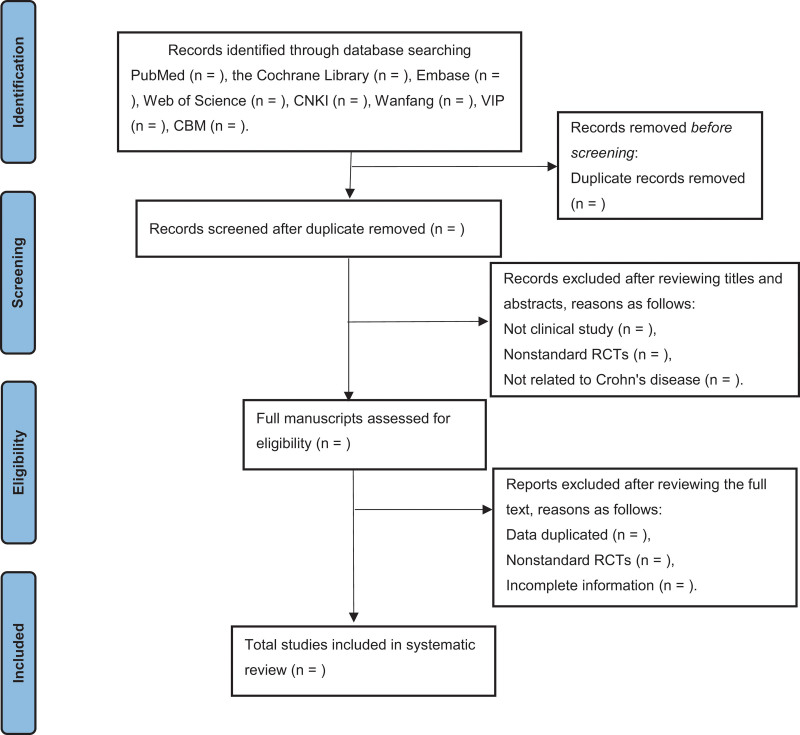
Flow chart of the search process.

To avoid duplicate studies being cited, we will use Note Express 3.2.0 software (http://www.inoteexpress.com/aegean/) for screening. Two independently reviewers will evaluate all citations retrieved in the above search strategy after reading the titles and abstracts. Upon completion then appropriate studies will be included by independent complete reading of the full text. In case of disagreement between the 2 reviewers, it will be referred to a third reviewer for discussion to reach consensus. Finally, all data will be extracted by the 2 reviewers according to the recommendations of the Cochrane Interventions Systematic Review Manual. The following data will be extracted: authors, age, region, number of participants, interventions, treatment duration, acupuncture points, outcome measures, adverse events, and relapse rates. The PRISMA flow chart of the study selection process is shown in Fig. 1.^[12]^

### 2.8. Additions of missing data

If information in an article is incomplete, we will attempt to contact the study authors by email or phone to obtain missing data or clarification. Otherwise, we will conduct with step-by-step exclusion of each study from the analysis based on the available information to determine the potential impact of the missing information on the results of the meta-analysis.

### 2.9. Risk of bias assessment

Two independently reviewers will evaluate the risk of bias by using the Cochrane Risk of Bias tool.^[13]^ We will document the method of random sequence generation and allocation concealment, whether participants, personnel and outcome assessments were blinded, the presence of wear and tear bias for incomplete outcome data, the presence of reporting bias for selective reporting, and the presence of other biases such as pre-sample size estimates and early trial stopping.

### 2.10. Data synthesis and statistical analysis

We will use Review Manager 5.4 software to complete the data analysis. Random or fixed effects models will be used for the meta-analysis. We will compare dichotomous outcomes between the groups using the risk ratio and continuous data using the mean difference and the corresponding standard deviation. We will summarize the risk ratio and mean difference with a 95% confidence interval (CI); if the 95% CI includes a value of 0 or 1 for continuous or dichotomous data, respectively, we will consider the difference to be statistically insignificant. We will interpret them using the following criteria: 25% of *I*^2^ values are considered as low heterogeneity level, 50% of *I*^2^ as moderate level and 75% as high level. Data will be analyzed using a fixed-effects model when low or moderate heterogeneity between studies is present, while a random-effects model will be used to analyze data when high heterogeneity is present (*P* < .05, *I*^2^ > 50%), and heterogeneity will be corrected by subgroup analysis.

### 2.11. Additional analysis

If enough data is acquired and the relevant analysis is available and possible, we will conduct subgroup analyses in terms of the following factors.

1. The specific effects of interventions, age, duration and course of treatment on outcomes
2. Different acupuncture techniques (e.g., manual acupuncture, electroacupuncture, acupressure).
3. Different assessment points of main outcomes.

Correlation analysis is applied by using the SPSS modeler to apply the Apriori algorithm with different acupuncture points and specific intervention parameters in all study results and perform complex network presentation through Gephi to explore the most commonly used clinical prescriptions for acupuncture for CD.

### 2.12. Reporting bias analysis

The reporting bias will be assessed by funnel plots. Additionally, Begg’s test and Egger’s test will be utilized to determine if the funnel plot is symmetrical.

### 2.13. Confidence in cumulative evidence

We will use the Grading of Recommendations Assessment, Development and Evaluation (GRADE) Analyzer Guideline Development Tool to assess the confidence level of effect estimates for each outcome.^[14]^ Evidence quality will be independently assessed by 2 review authors based on 5 Grade rating criteria (study limitations, imprecision, inconsistency, indirectness, and publication bias). The quality of evidence will be categorized into 1 of 4 possible ratings (high, moderate, low, and very low). Any disagreements that arise will be resolved by consensus or consultation with the third review author.

## 3. Discussion

In China, acupuncture has been widely used in the clinical treatment of inflammatory bowel disease,^[7]^ especially for CD, and is believed to play a significant role in reducing the expression of inflammatory factors in patients with CD.^[8–10,15–17]^ The World Health Organization in its Traditional Medicine Strategy 2014–2023 advocates an evidence-based approach to traditional, complementary alternative and integrative medicine.^[18]^ Systematic reviews and meta-analyses are considered as evidence-based research assessments for evaluating the efficacy of clinical interventions. Although a systematic review is essential for evaluating the efficacy of acupuncture on CD, a recent survey of studies have shown that the overall quality of most acupuncture systematic reviews methodologies was with low quality.^[19]^ A standard protocol is needed prior of the systematic review and meta-analysis. While a relative high-quality systematic reviews and meta-analyses can effectively assist in decision making based on a reliable, clear, and comprehensive synthesis of the best available evidence for a given clinical problem.^[20]^ In contrast, low-quality systematic reviews and meta-analyses may be prone to biased conclusions due to the presence of inappropriate literature searches, lack of critical appraisal of the absence of included original studies, and high heterogeneity,^[21,22]^ which may instead mislead decision makers in their clinical practice.^[23]^ Therefore, considering this current situation, this protocol outlines the planned scope and methods of an upcoming systematic review and meta-analysis of the efficacy and safety of acupuncture for CD, and is the first protocol for a systematic review and meta-analysis of the efficacy and safety of acupuncture for CD, with key indicators including clinical efficacy, symptom improvement, quality of life, and adverse effects, providing a rigorous assessment of the effectiveness and safety of acupuncture for CD, and it is also expected to provide new research treatment guidance decisions for acupuncture for CD. In addition, we will conduct a subgroup analysis of the clinical trial studies of acupuncture for CD using different acupuncture methods, acupuncture points, and related acupuncture treatment parameters in order to provide a valuable reference for the clinical treatment protocol setting of acupuncture for CD.

## Author contributions

**Conceptualization:** Jiazhen Cao, Min He.

**Data curation:** Qianhui Yu, Renming Liu.

**Formal analysis:** Jiazhen Cao, Renming Liu.

**Funding acquisition:** Tie Li.

**Investigation:** Qianhui Yu, Mengmeng Sun.

**Methodology:** Jiazhen Cao, Qianhui Yu.

**Project administration:** Li Tie.

**Resources:** Jiazhen Cao, Renming Liu.

**Software:** Jiazhen Cao, Qianhui Yu.

**Supervision:** Fuchun Wang, Mengmeng Sun.

**Validation:** Wu Liu.

**Visualization:** Fuchun Wang, Min He.

**Writing** – **original draft:** Jiazhen Cao.

**Writing** – **review and editing:** Fuchun Wang, Tie Li.
